# Natural and controlled ovulation in South American camelids

**DOI:** 10.21451/1984-3143-AR2018-0033

**Published:** 2018-08-03

**Authors:** Gregg P. Adams, Marcelo H. Ratto, Rodrigo A. Carrasco

**Affiliations:** 1 Veterinary Biomedical Sciences, University of Saskatchewan, Saskatoon, SK Canada; 2 Department of Animal Science, Universidad Austral de Chile, Valdivia, Chile

**Keywords:** Camelid, ovary, ovulation, superovulation, synchronization.

## Abstract

The four species of New World camelids and 2 species of Old World camelids derived from a common ancestor in North America. The reproductive characteristics, particularly those involving ovarian function and ovulation, are remarkably similar among the 6 living species of camelids, so much so that interspecies hybrids of nearly all possible combinations have been documented. Camelids are induced-ovulators, triggered by an ovulation-inducing factor in seminal plasma. The timing and mechanism of endocrine events leading to ovulation are discussed, as well as the discovery, identification and mode of action of the seminal factor responsible. The applied aspects of our present understanding are discussed with specific reference to controlled induction of ovulation, ovarian synchronization, and superovulation. Emphasis has been given to the literature on llamas and alpacas, with some reference to studies done in wild species of South American camelids and Old World camels.

## Introduction

No large group of recently extinct placental mammals remains as evolutionarily cryptic as the approximately 280 genera grouped as ‘South American native ungulates’ (Welker *et al*., 2015). This is particularly true of the phylogeny and taxonomy of the family Camelidae. The crown family Camelidae originated in North America 40 to 45 million years ago (Ma) and evolved into two tribes, the Camelini (Old World camels) and Aucheniini (or Lamini, New World camelids) 11 to 17 Ma (Stanley *et al*. 1994; Heintzman *et al*. 2015). Recent paleogenomic data suggest that 2 major groups of the Camelini tribe, *Camelops* and *Paracamelus* gave rise to present day Camelus species (dromedary and Bactrian camels) after migrating to Asia across the Bering landbridge from 7 to 5 Ma. It is unclear if these 2 groups co-habited North America since *Paracamelus* died out in North America ~1 Ma, and *Camelops* (the largest of the ancestral camelids) died out in North America ~13 thousand years ago along with other of the Pleistocene megafauna (Heintzman *et al*., 2015).

Contrary to the previous notion of having descended from *Paleolama*, New World (or South American) camelids likely descended from another branch of the Aucheniini (Lamini) tribe called *Hemiauchenia* between 9-11 Ma (Wheeler, 1995; Scherer, 2013). After migration from North to South America across the Panamanian isthmus beginning ~2.7 Ma, only 2 genera (*Lama* and *Vicugna*) survived the end of the Pleistocene period ~10 thousand years ago, and only those in South America (Wheeler *et al*., 1995; Heintzmen *et al*., 2015). The Inca and Aymara empires of pre-colonial South America began domesticating South American camelids ~6 to 7 thousand years ago. However, as a result of severe population bottle-necks in both genera beginning at the time of the Spanish Conquest in 1532, and subsequent hybridization of domestic lines, the lineage of today’s domestic species (alpaca and llama) is equivocal (Stanley *et al*., 1994). Currently, the domestic llama (*Lama glama*) is thought to have descended from the wild guanaco (*Lama guanicoe*) and the domestic alpaca (*Vicugna pacos*) from the wild vicuna (*Vicugna vicugna;* Wheeler *et al*., 1995).

All four species of New World camelids are capable of interbreeding and producing fertile offspring without apparent reduced fecundity, as are the two species of Old World camels (reviewed in Skidmore *at al*., 2001). Despite geographic separation for at least the last 11 million years, hybridization between Old and New World camelids has also been documented through the use of artificial insemination and transfer of hybrid embryos (Skidmore *et al*.,1999, 2001). Fecundity of Old x New World crosses, however, is very low. Of 102 artificial inseminations, pregnancy was detected in only 9 (9%) and only 1 live offspring was born (1%). While all camelid species have the same number of chromosomes (2n = 74), the genetic distance between Old and New world camelids is apparently sufficient to make the pairing of homologous chromosomes no longer possible.

The purpose herein is to provide an overview on what is known about ovulation in camelids. We’ve included a discussion of historical and contemporary studies on the nature and mechanism of ovulation, the role of ovulation-inducing factor (OIF) in semen, and implications for controlled induction of ovulation, ovarian synchronization, and superovulation. Emphasis has been given to the literature on llamas and alpacas, with some reference to studies done in wild species of South American camelids and Old World camels.

## Camelids are induced ovulators

Gonadotropin releasing hormone (GnRH) is the central hypothalamic regulator of LH pulses in both spontaneous and induced ovulators. GnRH is produced by the hypothalamic neurons from a precursor polypeptide after enzymatic processing and then it is packaged in storage granules that are transported down neural axons to the median eminence (Fink, 1988). Immunocytochemical studies have indicated that GnRH neurons are scattered throughout the medio-basal hypothalamus (MBH), rostrally and dorsally to the preoptic area and ventromedial hypothalamic nuclei (Karsch, 1987). Although there are clear species differences in the localization of GnRH neurons, differences have not been related to the type of ovulation mechanism observed in domestic animals.

Spontaneous ovulators (cattle, sheep, horses, pig) have ovarian cycles where the periodic preovulatory LH surge and ovulation occur at regular intervals, controlled by the feedback effects of ovarian steroids on the pituitary gland and hypothalamus (Karsch, 1987; Turzillo and Net, 1999). In contrast, induced ovulators such as South American camelids do not have an ovarian cycle that is punctuated by regular periodic ovulation. Instead, copulation has been considered the required stimulus to induce ovulation (San Martin *et al*., 1968; England *et al*., 1969). In an early study designed to determine factors associated with eliciting ovulation in alpacas (Fernandez-Baca *et al*., 1970a), ovulation rate was compared among females that 1) were unmated, 2) were mounted only followed with or without artificial insemination, 3) had interrupted mating, 4) had sterile mating (vasectomized male) followed with or without artificial insemination, 5) had single or multiple uninterrupted mating (intact male), or 6) were given hCG. A high ovulation rate (80 to 100%) was observed in females mated by intact or vasectomized males or when hCG was given.

## Timing of endocrine and ovarian events

In the first endocrine study to correlate circulating concentrations of LH with mating and ovulation in camelids (Bravo *et al*., 1990), LH increased at 15 min, peaked at 2 h, and declined to basal levels by 7 h after natural mating. The rapid increase in plasma LH after mating in llamas resembled that observed in rabbits after a single mating (Jones *et al*., 1976). Apparently, the number of matings did not increase either the ovulation rate in alpacas (Fernandez-Baca *et al*, 1970a) or the amplitude of the LH surge in llamas and alpacas (Bravo *et al*., 1992), in contrast to other induced ovulators such as the cat in which multiple mating increased both plasma LH amplitude and ovulation rate (Concannon *et al*., 1980).

The interval from stimulus to ovulation was not reported in the study by Fernandez-Baca *et al*. (1970a) because ovaries were collected from a slaughterhouse 3 days after treatment. In a study using one-time examination of the ovaries during necropsy at 2 to 6 h intervals after mating (1-5 alpacas/time interval; San Martin *et al*., 1968), ovulation had occurred as early as 26 h after mating (3/5 alpacas) but the mean interval to and distribution of ovulations was not reported. In a study using one-time laparoscopic examination of the ovaries of alpacas at 12 h intervals after mating (Sumar *et al*., 1993), the interval to ovulation was 30 to 72 h in 50% (38/76) and approximately 30 h in 24% (18/76) of females.

Incorporation of B-mode ultrasonography into studies of ovarian function enabled rapid advancement in our understanding of follicular and luteal dynamics and factors associated with ovulation in camelids. A wave-like pattern of ovarian follicular development has been documented in llamas (Adams *et al*., 1990), alpacas (Vaughan *et al*., 2004), vicunas (Aba *et al*., 2005) and guanacos (Riveros *et al*., 2010). The wave pattern was characterized by periodic increases (every 15 to 20 days) in the number of antral follicles and the selection of a single dominant follicle of ≥7 mm in both llamas and alpacas (Adams *et al*., 1990; Vaughan *et al*., 2004). It has been proposed that the ability of llamas and alpacas to ovulate in response to a mating stimulus is influenced by the developmental status of the dominant follicle at the time of mating (Adams *et al*., 1990). Based on daily ultrasonography of the ovaries in llamas, spontaneous ovulation occurred in 2 of 25 (8%) unmated llamas and failure to ovulate occurred in 5 of 49 (10%) mated llamas (Adams *et al*., 1990). In separate studies, ovulation occurred on the second day after mating in 75% (6/8, Adams *et al*., 1989) and 96% (26/27, Adams *et al*., 1990) of llamas. Collectively, the interval from the first mating to ovulation was 2.0 ± 0.1 days, and was not affected by lactational status or the type of mating (vasectomized *vs*. intact male). By ultrasonographic examination every 4 h (Ratto *et al*., 2006), the interval to ovulation was 30.0 ± 0.5, 29.3 ± 0.6, and 29.3 ± 0.7 h in llamas given natural mating or treated with either 12.5 mg LH or 50 ug GnRH analogue (gonadorelin acetate), respectively.

Changes in plasma progesterone concentration have been characterized after treatment with hCG to induce ovulation (Fernandez-Baca *et al*., 1970b; Adam *et al*., 1989), after mating with a vasectomized male (Sumar *et al*., 1988) or an intact male (Bravo *et al*., 1990, 1991), and throughout pregnancy (Leon *et al*., 1990). In a study involving daily examination and blood sampling (Adams *et al*., 1991), measurement of the diameter of the corpus luteum by transrectal ultrasonography was an accurate method of assessing luteal function (plasma progesterone concentration) in llamas (r = 0.83, P < 0.0001). Corpora lutea were not detected and plasma progesterone concentration did not exceed 0.4 ng/ml in anovulatory (nonmated) llamas. In ovulatory nonpregnant (vasectomy-mated) llamas, the corpus luteum reached maximal diameter (12.8 mm) on mean day 7 (day 0 = ovulation), and regressed between days 10 and 12. In pregnant llamas, luteal diameter continued to increase until mean day 21 (16.3 mm); maximal diameter was maintained for the remainder of the observational period (day 60). Similarly, the corpus luteum in alpacas reached a maximum diameter of 14 mm 8 to 9 days after mating, and regressed 8 to 12 days after mating. Maximum plasma progesterone concentrations in nonpregnanct alpacas and vicunas occurred at 7 to 8 days post mating (Sumar *et al*., 1988; Aba *et al*., 1995).

## Ovulation-inducing factor in semen

### Discovery

Studies in China on Bactrian camels were the first to report an ovulation-inducing effect of seminal plasma (reviewed in Adams *et al*., 2016). At the time, this finding was largely dismissed in favor of the established notion that the trigger for induced ovulation is coital stimulation. During the same time period, results of studies in pigs (spontaneous ovulators) revealed that infusion of semen in the uterus of sows increased litter size (Murray *et al*., 1983). Thus, the concept that seminal plasma may have direct effect on hypothalamic-hypophyseal-gondal axis of the female began to take root. Confirmation of the existence of an ovulation-inducing factor (OIF) in semen came 20 years later in a series of studies done in llamas and alpacas where the intramuscular administration of seminal plasma induced ovulation in a high proportion of females (Adams, 2005).

Paradoxically, intrauterine infusion of seminal plasma induced ovulation in llamas at a lower rate than intramuscular administration. However, the addition of endometrial curettage (mimicking copulatory mucosal erosion) with intrauterine administration of seminal plasma resulted in a marked increase in ovulatory response, and supported the hypothesis that the effect of OIF is mediated by absorption into systemic circulation of the female (i.e., increased by endometrial hyperemia; Ratto *et al*., 2005). Intrauterine infusion of a larger dose of OIF (i.e., commensurate with the amount present in a normal ejaculate) resulted in a 100% of ovulation rate in llamas (Silva *et al*., 2015). Irrespective of the route of administration, seminal plasma (or purified OIF) elicited a surge in circulating concentrations of LH, demonstrating that the effect is mediated centrally at the hypothalamic-pituitary unit. Despite having branched from other artiodactyls more than 45 Ma, camelids have interesting features in common with spontaneous ovulators. One is that while rising concentrations of estradiol do not trigger the preovulatory LH surge in camelids, it appears that estradiol modulates the LH secretory response to OIF (Silva *et al*., 2012a). More surprisingly, OIF has been detected in the seminal plasma of every spontaneous ovulator tested to date (reviewed in Adams *et al*., 2016), suggesting the existence of as yet unknown pathways influencing reproduction.

### Identification

Chemical identification of OIF began by treating seminal plasma in ways intended to neutralize specific constituents and thereby ablate the ovulatory effect (reviewed in Adams *et al*., 2016). Treatment by heating (65ºC), charcoal-dextran, or proteinase K (38ºC for an hour) did not abolish the ovulation inducing effect of llama seminal plasma. However, treatment with pronase E, a complex mixture of proteases, ablated the ovulatory effect, suggesting that the molecule responsible of ovulation induction was in fact a protein. The use of a two-step chromatographic procedure allowed the isolation of a protein fraction that retained the ovulatory effect. The final identification of OIF was discovered during crystallography studies in which the amino acid sequence and protein structure of OIF was identical to the known neurotrophin beta-nerve growth factor (bNGF; Ratto *et al*., 2012). Similar chromatographic procedures have led to the identification and purification of OIF/bNGF in camel seminal plasma (Kumar *et al*., 2013) and its role in camel ovulation (Fatnassi *et al*., 2017).

### Luteotrophic effect

Additionally, OIF has been shown to have a powerful luteotrophic effect. Plasma progesterone concentration on day 7 after ovulation induced by OIF were 2.5 times higher than in GnRH treated animals (Adams *et al*., 2005), and luteal function was enhanced independent of follicle size at the time of treatment with OIF (Silva *et al*., 2014). A mechanistic association between NGF and angiogenic factors has been reported in the rat ovary and human granulosa cells (Julio-Pipper *et al*., 2006, 2009). An angiogenic mechanism was implicated in more recent studies of the luteotrophic effect of OIF/NGF in which the preovulatory follicle of OIF-treated llamas displayed a transient increase in vascularity 4 h after treatment, and corpus luteum vascularity was greater at day 6 after treatment compared to GnRH-treated llamas (Ulloa-Leal *et al*., 2014). Further, OIF treatment in llamas was associated with a 3-fold increase in the mRNA of steroidogenic enzymes in the corpus luteum at day 4 after ovulation compared to llamas induced to ovulate with GnRH (Silva *et al*., 2017).

The luteotrophic effect has been attributed largely to the increased amounts of LH secreted under OIF stimulation (Adams *et al*., 2005), and dose- response effect on ovulation rate, LH secretion, and corpus luteum function in llamas (Tanco *et al*., 2011). Interestingly, a similar dose-response effect on LH secretion was observed with increasing doses of GnRH in llamas, but luteal function (e.g. progesterone concentration) did not differ at different GnRH dosages (Silva *et al*., 2012b). Perhaps then, the requirements for luteal development are fully met by minimal GnRH doses, but the administration of OIF provides an extra signal that promotes luteogenesis. This view is consistent with several reports of high- and low-affinity receptors for NGF in the ovaries of different species (Dissen *et al*., 1996, 2000; Levanti *et al*., 2005; Carrasco *et al*., 2016).

### Proposed mechanism

It is well established that OIF stimulates LH secretion prior to ovulation in camelids, interacting directly or indirectly with the pituitary gland (reviewed in Adams *et al*., 2016). Consistent with an early report using rat pituitary cells and alpaca seminal plasma (Paolichi *et al*., 1999), treatment of primary cultures of llama and bovine pituitary cells with OIF induced LH secretion into the culture media (Bogle *et al*., 2012).

However, OIF treatment in vivo was not associated with a detectable rise in plasma LH or ovulation in prepubertal heifers (Tanco *et al*., 2012), nor in llamas pre-treated with a GnRH receptor blocker (Cetrorelix; Silva *et al*., 2011). These findings support the hypothesis that OIF acts upstream from the pituitary, most likely at the level of the hypothalamus, perhaps on GnRH neurons themselves. For this hypothesis to be plausible, OIF must cross the blood-brain-barrier and neurons in the hypothalamus must express NGF receptors. In mice, NGF has been shown to cross the blood-brain-barrier (Pan *et al*., 1998); however, the site and the mechanism by which it crosses remains unknown. In an immunofluorescent study to determine if llama GnRH neurons possess receptors for NGF (trkA and p75; high- and low-affinity receptors, respectively), we found no co-localization with GnRH neurons (Carrasco RA et al., 2018; Veterinary Biomedical Sciences, University of Saskatchewan, Saskatoon, SK, Canada; submitted paper), suggesting that the neuronal target for OIF in the hypothalamus is a group of interneurons that synapses with GnRH neurons, stimulating its secretion into the portal system.

### Ovulation synchronization

Experiments involving the empirical use of progesterone have been reported in llamas and alpacas, based on studies done in cattle and sheep. The rationale for using progesterone alone to synchronize follicular development in camelids, however, is unclear since regular luteal phases are not a characteristic of the ovarian pattern in camelids (i.e. induced ovulators) and follicular waves continue to emerge at regular intervals during progestational states (i.e. after sterile mating or during pregnancy; Adams *et al*., 1990). Induction of a luteal phase was associated with a decrease in the diameter profile of the dominant follicle and a shorter interval between follicular waves (Adams *et al*., 1990). Similarly, the use of an intravaginal progesterone- releasing device in llamas (Chaves *et al*., 2002) and vicunas (Aba *et al*., 2005) resulted in a decrease in the maximum diameter of the dominant follicle, but no data were reported regarding the emergence of a new follicular wave. In a controlled synchronization study (Ratto *et al*., 2003), llamas were treated with saline (control), a combination of estradiol plus progesterone, LH, or by transvaginal ultrasound-guided follicle ablation (n = 20 per group). Compared to controls, treatment with LH or follicle ablation were equally effective at synchronizing and shortening the interval to follicular wave emergence to 2 days after treatment, and to the day on which the new dominant follicle reached ≥7 mm (ovulatory capability; 5 days after treatment), while the steroid-treated group was intermediate in effect. Compared to controls, synchronization treatment resulted in a higher pregnancy rate to a single, timed mating (54 *vs*. 76%; Ratto *et al*., 2003).

### Superovulation

Important limitations to implementing embryo transfer technology in South American camelids include an inconsistent ovarian superstimulatory response, the challenge of collecting and processing semen, low embryo collection efficiency, and the recovery of advanced-stage embryos (hatched blastocyst) that are difficult to handle and cryopreserve. Studies have involved superstimulatory treatments with FSH or eCG during the follicular or sexual receptivity phase or during natural or artificially induced luteal phases (review in Ratto *et al*., 2013). The superovulatory response in camelids, estimated by the total number of corpora lutea on the day of embryo collection, ranges from 0 to 17 per female, with an embryo recovery rate ranging from 0 to 45% (Del Campo *et al*., 1995; Ratto *et al*., 2013). In a retrospective analysis of 5547 single- or multiple-ovulation embryo transfers performed on commercial alpaca farms in Australia (Vaughan *et al*., 2013), factors found to have a significant impact on the success of embryo transfer were the day of flushing after mating (8 and 9 days after mating were best), embryo diameter (larger were better), embryo quality, day of transfer to recipients (7 and 8 days after GnRH were best), and the age of the recipient (≤15 years). Compared to single-ovulation donors, superovulation of donors resulted in an average of 6.4 ovulations and 3.6 times as many transferrable embryos (0.67 *vs*. 2.44) and offspring (0.29 *vs*. 1.02) per donor flushed. These results are in agreement with an earlier controlled study (Huanca *et al*., 2009) in which superstimulatory treatment with eCG (with or without progestin) induced an average of 9.3 ovulations and 4.3 embryos per donor flushed; 10.1 times as many ovulations and 5.9 times as many embryos as in unstimulated controls.

Although not critically examined in camelids, studies in cattle have documented the positive effect of initiating ovarian superstimulatory treatment at the time of follicular wave emergence (reviewed in Adams, 1994 and Adams *et al*., 2012). With successful development of protocols to control follicle development and ovulation, superstimulation may now be initiated at a pre-scheduled time to optimize the ovulatory response. Ovarian superstimulation with either FSH or eCG given during follicular wave emergence induced by follicle ablation were equally efficacious in inducing multiple follicle growth in llamas without progesterone/progestin treatment (Ratto *et al*., 2004). Similarly, eCG (with or without progestin) given to llamas at the time of follicular emergence induced by LH administration resulted in an average 8.6 and 10.1 corpora lutea (eCG with and without progestin, respectively) and an average of 3.7 and 4.9 embryos, respectively (Huanca *et al*., 2009).

## Other camelid species

Most of studies on the reproductive physiology of South American Camelids have been conducted in the domestic species, llamas and alpacas, and results may not necessarily be extrapolated to the wild species. A wave-like pattern of ovarian follicular development similar to that described for llamas and alpacas has been reported in vicuñas (Aba *et al*., 2005) and guanacos (Riveros *et al*., 2010). The maximum diameter of the dominant follicle was 8.9 ± 0.9 mm (range: 6.2-11.2) and 10.2 ± 2.1 mm (range: 7.2-16.1 mm) for vicuñas and guanacos, respectively. In another vicuña study (Miragaya *et al*., 2004), intramuscular administration of 750 IU of eCG with or without an intravaginal progesterone device induced the growth of 8 to 13 follicles ≥6 mm per female. No studies have been conducted in the wild species on the timing of ovulation after natural mating or administration of GnRH, hCG, or OIF.

The Old World camelids, dromedary and Bactrian camels, inhabit the dry desert of Africa, Arabia and the cold regions of China and Mongolia, respectively. They are seasonal breeders, induced ovulators, and also display a wave-like pattern of ovarian follicular development (Skidmore, 2011). The maximum diameter of the dominant follicle was 2.0 ± 0.1 cm (range: 1.5- 2.5) in both dromedary and Bactrian camels; however, in about 50% of follicular waves in dromedaries the dominant follicle reached a maximum diameter of 4.0-6.4 cm. The diameter of the follicle on the day before ovulation was 1.3 ± 0.2 cm in diameter and ovulation was detected between 28 and 36 h after mating (Skidmore, 2011). A GnRH agonist, Buserelin, or 3000 IU of hCG have been used to induce ovulation or synchronize follicular wave emergence in camels. Treatment with GnRH or hCG induced ovulation in 85% of dromedary camels when given in the presence of a preovulatory follicle between 1.0 and 1.9 cm, but was ~12% when given in the presence of a follicles between 2.0 and 2.9 cm, and none ovulated when treatment was given when the largest follicle was ≤0.9 or ≥3.0 cm in diameter (Skidmore *et al*., 1996). In a more recent study in dromedaries, two GnRH injections 14 days apart, with or without PGF2a 7 days after the first GnRH treatment, were effective methods of synchronizing wave emergence and subsequent ovulation (Skidmore et al., 2009), but the efficacy of synchronization for fixed-time insemination has not been reported.

## Conclusions

Much has been learned in the last 20 years about ovarian function in camelids. As the largest domestic species of induced ovulator, llamas, alpacas and camels have provided an opportunity to re-examine our understanding of factors and mechanisms involved in ovulation. Studies involving serial examination of ovarian and endocrine events permitted testing new hypotheses about the role of semen in these and other species of induced ovulators, as well as in spontaneous ovulators. The discovery of a factor in semen that has a direct effect on the hypothalamo-pituitary axis of the inseminated female is new and exciting, and may have broad implications. Basic and applied studies are on- going in the hope of elucidating the precise site of action, and neuro-endocrine cells involved in initiating the preovulatory surge in LH. A better understanding of ovarian follicular dynamics and treatments designed to control follicle growth and ovulation have enabled the use of reproductive techniques such as synchronization and timed-insemination, and have made viable the business of embryo transfer in both Old and New World camelids.

## References

[B1] Aba MA, Forsberg M, Kindhal H, Sumar J, Edqvist L (1995). Endocrine changes after mating in pregnant and non-pregnant llamas and alpacas. Acta Vet Scand.

[B2] Aba MA, Miragaya MH, Chaves MG, Capdevielle EF, Rutter B, Aguero A. (2005). Effect of exogenous progesterone and eCG treatment on ovarian follicular dynamics in vicuñas (Vicugna vicugna). Anim Reprod Sci.

[B3] Adam CL, Moir CE, Shiach P. (1989). Plasma progesterone concentrations in pregnant and nonpregnant llamas (Lama glama). Vet Rec.

[B4] Adams GP, Griffin PG, Ginther OJ. (1989). In situ morphologic dynamics of ovaries, uterus, and cervix in llamas. Biol Reprod.

[B5] Adams GP, Sumar J, Ginther OJ. (1990). Effects of lactational and reproductive status on ovarian follicular waves in llamas (lama glama). J Reprod Fertil.

[B6] Adams GP, Sumar J, Ginther OJ. (1991). Form and function of the corpus luteum in llamas. Anim Reprod Sci.

[B7] Adams GP. (1994). Control of ovarian follicular waves dynamics in cattle: implications for synchronization and superstimulation. Theriogenology.

[B8] Adams GP, Ratto MH, Huanca W, Singh J. (2005). Ovulation - inducting factor in the seminal plasma of llamas and alpacas. Biol Reprod.

[B9] Adams GP, Singh J, Baerwald AR. (2012). Large animal models for the study of ovarian follicular dynamics in women. Theriogenology.

[B10] Adams GP, Ratto MH, Silva ME, Carrasco RA. (2016). Ovulation-inducing factor (OIF/NGF) in seminal plasma: a review and update. Reprod Domest Anim.

[B11] Bogle OA, Ratto MH, Adams GP. (2012). Ovulation- inducing factor induces LH secretion from pituitary cells. Anim Reprod Sci.

[B12] Bravo PW, Fowler ME, Stabenfeldt GH. (1990). Endocrine response in the llama to copulation. Theriogenology.

[B13] Bravo PW, Stabenfeldt GH, Lasley BL, Fowler ME. (1991). The effect of ovarian follicle size on pituitary and ovarian responses to copulation in domesticated South American camelids. Biol Reprod.

[B14] Bravo PW, Stabenfeldt GH, Fowler ME, Lasley BL. (1992). Pituitary response to repeated copulation and/or gonadotropin-releasing hormone administration in llamas and alpacas. Biol Reprod.

[B15] Carrasco R, Singh J, Adams GP. (2016). The dynamics of trkA expression in the bovine ovary are associated with a luteotrophic effect of ovulation-inducing factor/nerve growth factor (OIF/NGF). Reprod Biol Endocrinol.

[B16] Chaves MG, Aba MA, Aguero A, Egey J, Berestin V, Rutter B. (2002). Ovarian follicular wave pattern and the effect of exogenous progesterone on follicular activity in non-mated llamas. Anim Reprod Sci.

[B17] Concanon P, Hodgson B, Lein D. (1980). Reflex LH release in estrous cats following single and multiple copulations. Biol Reprod.

[B18] Del Campo MR, Del Campo CH, Adams GP, Mapletoft RJ. (1995). The application of new reproductive technologies to South American camelids. Theriogenology.

[B19] Dissen GA, Hill DF, Costa ME, Les Dees CW, Lara HE, Ojeda SR. (1996). A role for trkA nerve growth factor receptors in mammalian ovulation. Endocrinology.

[B20] Dissen GA, Parrott JA, Skinner MK, Hill DF, Costa MA, Ojeda SR. (2000). Direct effects of nerve growth factor on thecal cells from antral ovarian follicles. Endocrinology.

[B21] England BG, Foot WC, Matthews DH, Cardozo AG, Riera S. (1969). Ovulation and corpus luteum function in the llama (lama glama). J Endocrinol.

[B22] Fatnassi M, Cebrian-Perez JA, Salhi I, Perez-Pe R, Seddik MM, Casao A, Khorchani T, Muiño-Blanco T, Hammadi M. (2017). Identification of β-nerve growth factor in dromedary camel seminal plasma and its role in induction of ovulation in females. Emir J Food Agric.

[B23] Fernandez-Baca S, Hansel W, Novoa C. (1970a). Corpus luteum function in the alpaca. Biol Reprod.

[B24] Fernandez-Baca S, Madden DHL, Novoa C. (1970b). Effect of different mating stimuli on induction of ovulation in the alpaca. J Reprod Fertil.

[B25] Fink G., Knobil E, Neill J (1988). The Physiology of Reproduction.

[B26] Heintzman PD, Zazula GD, Cahill JA, Reyes AV, MacPhee RDE, Shapiro B. (2015). Genomic data from extinct North American Camelops revise camel evolutionary history. Mol Biol Evol.

[B27] Huanca W, Cordero A, Huanca T, Cardenas O, Adams GP, Ratto MH. (2009). Ovarian response and embryo production in llamas treated with eCG alone or with progestin vaginal sponges at the time of follicular wave emergence. Theriogenology.

[B28] Jones EF, Bain JB, Odell WD. (1976). Postcoital luteinizing hormone release in male and female rabbits as determined by radioimmunoassay. Fertil Steril.

[B29] Julio-Pipper M, Lara H, Bravo JA, Romero C. (2006). Effects of nerve growth factor (NGF) o blood vessels area and expression of the angiongenic factors VEGF and TGFbeta 1 in the rat ovary. Reprod Biol Endocrinol.

[B30] Julio-Pipper M, Lozada P, Tapia V, Vega M, Miranda M, Vantman D, Ojeda SR, Romero C. (2009). Nerve growth factor induces vascular endotelial growth factor expression in granulosa cells via a TrkA receptor/mitogen-activated protein kinase- extracelullarly regulated kinase 2-dependent pathway. J Clin Endocrinol Metab.

[B31] Karsch FJ. (1987). Central actions of ovarian steroids in the feedback regulation of pulsatile secretion of luteinizing hormone. Ann Rev Physiol.

[B32] Kumar S, Sharma, VK, Singh S, Hariprasad GR, Mal G, Srinivasan A, Yadav S (2013). Proteomic identification of camel seminal plasma: purification of β-nerve growth factor. Anim Reprod Sci.

[B33] Leon JB, Smith BB, Timm KI, Le Cren G. (1990). Endocrine changes during preganancy, parturition and the early post-partum period in the llama (Lama glama). J Reprod Fertil.

[B34] Levanti MB, Germaná A, Abbate F, Montalbano G, Vega JA, Germaná G. (2005). TrkA and p75NTR in the ovary of adult cow and pig. J Anat.

[B35] Miragaya MH, Aba MA, Capdevielle EF, Ferrer MS, Chaves MG, Rutter B, Aguero A. (2004). Follicular activity and hormonal secretory profile in vicuna (Vicugna vicugna). Theriogenology.

[B36] Murray FA, Grifo AP, Parker. CF (1983). Increased litter size in gilts by intrauterine infusion of seminal and sperm antigens before breeding. J Anim Sci.

[B37] Pan W, Banks WA, Kastin AJ. (1998). Permeability of the blood-brain barrier to neurotrophins. Brain Res.

[B38] Paolicchi F, Urquieta B, Del Valle L, Bustos- Obregon E. (1999). Biological activity of the seminal plasma of alpacas: stimulus for the production of LH by pituitary cells. Anim Reprod Sci.

[B39] Ratto MH, Singh J, Huanca H, Adams GP. (2003). Ovarian follicular wave synchronization and fixed-time natural mating in llamas. Theriogenology.

[B40] Ratto MH, Singh J, Huanca H, Adams GP. (2004). In vivo and in vitro maturation of llama oocyte. Theriogenology.

[B41] Ratto MH, Huanca W, Singh J, Adams GP. (2005). Local versus systemic effect of ovulation-inducing factor in the seminal plasma of alpacas. Reprod Biol Endocrinol.

[B42] Ratto MH, Huanca W, Singh J, Adams GP. (2006). Comparison of the effect of natural mating, LH, and GnRH on interval to ovulation and luteal function in llamas. Anim Reprod Sci.

[B43] Ratto MH, Leduc YA, Valderrama XP, Van Straten KE, Delbaere LT, Pierson RA, Adams GP. (2012). The nerve of ovulation-inducing factor in semen. Proc Natl Acad Sci USA.

[B44] Ratto MH, Silva EM, Huanca W, Huanca T, Adams GP. (2013). Induction of superovulation in South American Camelids. Anim Reprod Sci.

[B45] Riveros JL, Schuler G, Bonacic C, Hoffmann B, Chaves MG, Urquieta B. (2010). Ovarian follicular dynamics and hormonal secretory profiles in guanacos (Lama guanicoe). Anim Reprod Sci.

[B46] San Martin M, Copaira M, Zuniga J, Rodriguez R, Bustinza G, Acosta L. (1968). Aspects of reproduction in the alpaca. J Reprod Fertil.

[B47] Scherer CS. (2013). The Camelidae (Mammalia, Artiodactyla) from the quaternary of South America: cladistic and biogeographic hypotheses. J Mamm Evol.

[B48] Silva ME, Smulders JP, Guerra M, Valderrama XP, Letelier C, Adams GP, Ratto MH. (2011). Cetrorelix suppresses the preovulatory LH surge and ovulation induced by ovulation-inducing factor (OIF) present in llama seminal plasma. Reprod Biol Endocrinol.

[B49] Silva ME, Colazo MG, Ratto MH. (2012a). GnRH dose reduction decreases pituitary LH release and pituitary response but does not affect corpus luteum (CL) development and function in llamas. Theriogenology.

[B50] Silva ME, Recabarren MP, Recabarren SE, Adams GP, Ratto MH. (2012b). Ovarian estradiol modulates the stimulatory effect of ovulation-inducing factor (OIF) on pituitary LH secretion in llamas. Theriogenology.

[B51] Silva ME, Ulloa-Leal C, Norambuena C, Fernandez A, Adams GP, Ratto MH. (2014). Ovulation inducing factor (OIF/NGF) from seminal plasma origin enhances Corpus luteum formation in llamas regardles the preovulatory follicle diameter. Anim Reprod Sci.

[B52] Silva ME, Fernandez A, Ulloa-Leal C, Adams GP, Berland MA, Ratto MH. (2015). LH release and ovulatory response after intramuscular, intravenous, and intrauterine administration of β-nerve growth factor of seminal plasma origin in female llamas. Theriogenology.

[B53] Silva ME, Ulloa-Leal C, Valderrama XP, Bogle OA, Adams GP, Ratto MH. (2017). Nerve growth factor from seminal plasma origin (spβ-NGF) increases CL vascularization and level of mRNA expression of steroidogenic enzymes during the early stage of corpus luteum development in llamas. Theriogenology.

[B54] Skidmore JA, Billah M, Allen WR. (1996). The ovarian follicular wave pattern and induction of ovulation in the mated and non-mated one-humped camel (Camelus dromedaries). J Reprod Fertil.

[B55] Skidmore J A, Billah M, Binns M, Short R V, Allen W R (1999). Hybridizing Old and New World camelids: Camelus dromedarius x Lama guanicoe. Proc R Soc Lond B Biol Sci.

[B56] Skidmore JA, Billah M, Short RV, Allen WR. (2001). Assisted reproductive techniques for hybridization of camelids. Reprod Fertil Dev.

[B57] Skidmore JA, Adams GP, Billah M (2009). Synchronisation of ovarian follicular waves in the dromedary camel (Camelus dromedarius). Anim Reprod Sci.

[B58] Skidmore JA. (2011). Reproductive physiology in female old world camelids. Anim Reprod Sci.

[B59] Stanley HF, Kadwell M, Wheeler JC. (1994). Molecular evolution of the family Camelidae: a mitochondrial DNA study. Proc R Soc Lond B Biol Sci.

[B60] Sumar J, Fredriksson G, Alarcon V, Kindahl H, Edqvist LE. (1988). Levels of 15-keto-13, 14-dihydro- PFG2 alpha, progesterone and oestradiol-17 beta after induced ovulations in llamas and alpacas. Acta Vet Scand.

[B61] Sumar J, Bravo PW, Foote WC. (1993). Sexual receptivity and time of ovulation in alpacas. Small Rumin Res.

[B62] Tanco VM, Ratto MH, Lazzarotto M, Adams GP. (2011). Dose-response of female llamas to ovulation- inducing factor from seminal plasma. Biol Reprod.

[B63] Tanco VM, Van Steelandt MD, Ratto MH, Adams GP (2012). Effect of purified llama ovulation- inducing factor on ovarian function in cattle. Theriogenology.

[B64] Turzillo AM, Nett TM. (1999). Regulations of the GnRH receptor gene expression in sheep and cattle. J Reprod Fertil Suppl.

[B65] Ulloa-Leal C, Bogle OA, Adams GP, Ratto MH. (2014). Luteotrophic effect of ovulation-inducing factor/nerve growth factor present in the seminal plasma of llamas. Theriogenology.

[B66] Vaughan J, Macmillan KL, D’Occhio MJ. (2004). Ovarian follicular wave characteristics in alpacas. Anim Reprod Sci.

[B67] Vaughan J, Mihm M, Wittek T. (2013). Factors influencing embryo transfer success in alpacas - A retrospective study. Anim Reprod Sci.

[B68] Welker F, Collins MJ, Thomas JA, Wadsley M, Brace S, Cappellini E, Turvey ST, Reguero M, Gelfo JN, Kramarz A, Burger J, Thomas-Oates J, Ashford DA, Ashton PD, Rowsell K, Porter DM, Kessler B, Fischer R, Baessmann C, Kaspar S, Olsen JV, Kiley P, Elliott JA, Kelstrup CD, Mullin V, Hofreiter M, Willerslev E, Hublin JJ, Orlando L, Barnes I, MacPhee RD. (2015). Ancient proteins resolve the evolutionary history of Darwin’s South American ungulates. Nature.

[B69] Wheeler JC. (1995). Evolution and present situation of the South American Camelidae. Biol J Linn Soc.

